# An eco-psychological framework for research on the physical environment of childcare classrooms and children’s play behavior

**DOI:** 10.3389/fpsyg.2024.1463151

**Published:** 2024-12-17

**Authors:** Chenhao Deng, Zhiyi Zhao, Nur maizura Ahmad Noorhani, Arniatul Aiza Mustapha

**Affiliations:** ^1^College of Built Environment, Universiti of Technologi MARA, Shah Alam, Selangor, Malaysia; ^2^Department of Interior Design, College of Art and Design, Jiangxi Institution of Fashion Technology, Nanchang, Jiangxi Province, China; ^3^Center of Studies for Interior Architecture, Faculty of Architecture, Planning and Surveying, Universiti of Technologi MARA, Shah Alam, Selangor, Malaysia

**Keywords:** ecological psychology, affordance, behavior setting, theoretical framework, perception-action loop, child-environment interdependence

## Abstract

Ecological psychology is an approach focused on the perception and behavior of organisms and environments, offering psychological insights for research on children. This study primarily explored the concepts of affordance and behavior setting based on an eco-psychological perspective concerning children’s behaviors and environment. Through a review of previous studies, we differentiated that affordance theory emphasizes children’s direct perceptions of environmental functions, whereas the concept of behavior setting highlights the dyadic relationship between long-term behaviors and environmental material features. However, existing studies on child–environment interactions often overlook children’s immediate actions in the context of affordance theory and fail to account for the dynamic nature of behavior settings. By integrating the distinctive traits of both theories, this study proposes an anticipatory framework based on ecological psychology to guide research on children’s environments, particularly within the indoor spaces of childcare facilities. Future studies should investigate the connections within this framework through field studies of childcare center environments and observations of children’s actions and behaviors during free play to assess congruence with environmental affordances.

## 1 Introduction

Ecological psychology examines the study of perception, cognition, and behavior, emphasizing the intricate relationship between organisms and their environment, pioneered by J. J. Gibson in the realm of perceptual research and E. J. Gibson in the domain of developmental psychology in the 1950s. This theory presents an alternative viewpoint to cognitivism and behaviorism and provides a third way to understand cognition (Lobo et al., 2018). Gibson rejected the mainstream theories of perception based on the premise of stimulus poverty, subsequent physicalist stimulus conceptualization, and the passivity of the perceiver (Lombardo, 2017). Unlike environmental psychology, which emphasizes the multiple temporal scales of human–environment transactions (Gatersleben, 2018; Gifford, 2014; Stokols, 1978), ecological psychology emphasizes the real time and continuity of perception and action (Gibson, 1979; Raymond et al., 2017), treats the environment–organism system as a unit of analysis, and regards *affordance* as the main object for studying perception (Michaels and Palatinus, 2014). Correspondingly, this approach considers embodied, situated, and non-representational characteristics of perception (Lobo et al., 2018). Based on this, ecological psychology not only considers organisms and environments as separate entities but also sees them as inherently coupled and interdependent. Thus, perception is a meaningful environmental attribute or affordance unique to a perceiver’s capabilities and needs.

**Figure 1:**
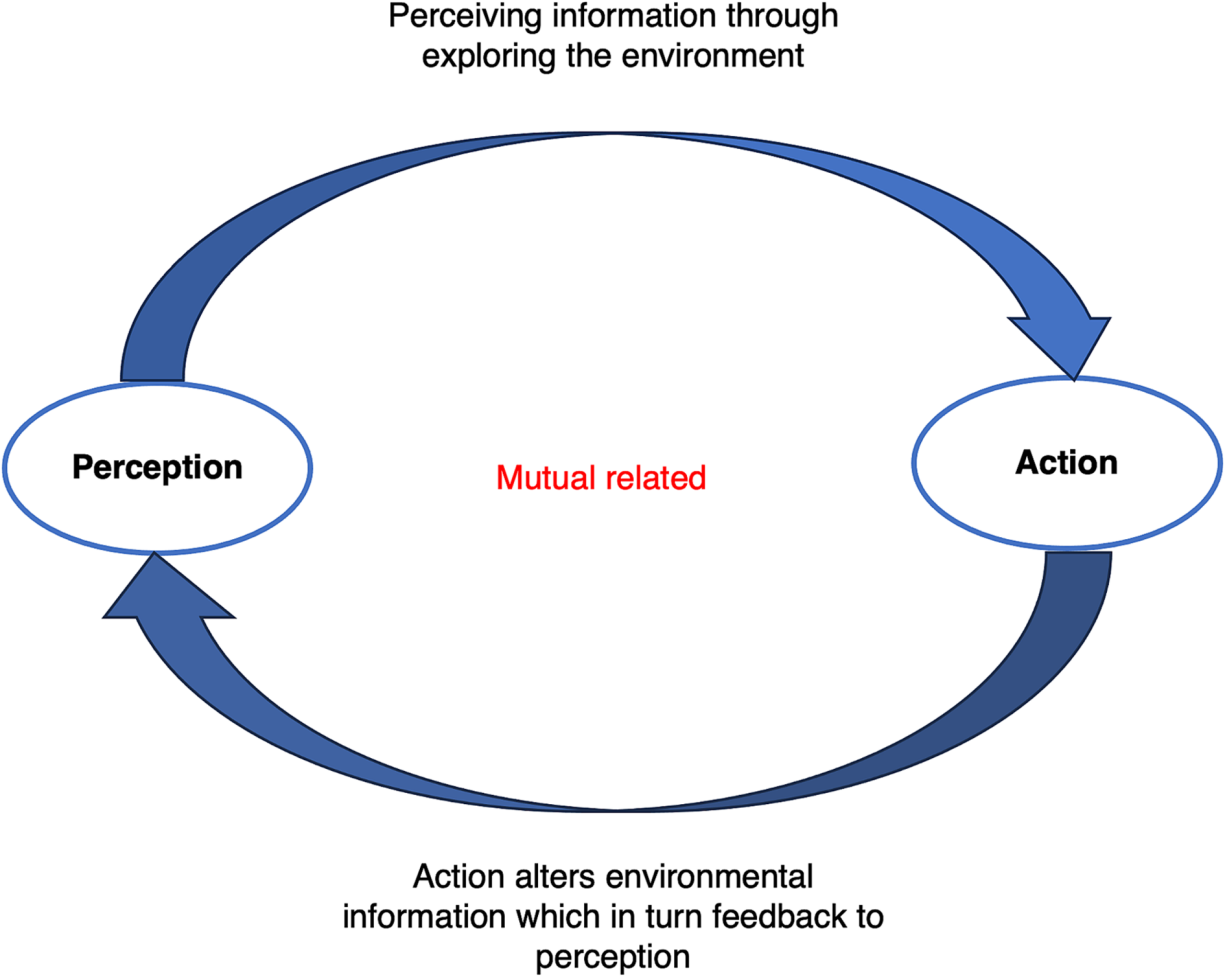
Mutual nexus between the perception and action of the organism which forms the perception–action loop.

One of Gibson’s posits in ecological psychology is direct perception, meaning that an organism’s perception is a direct process of collecting information from the surroundings, which, in turn, governs actions. This perception does not involve complex calculations or mental representations; rather, it is shaped by the cognition, intentions, and physical capabilities of the perceiver (Raymond et al., 2017). When children interact with the environment, the distinctive characteristics of the physical environment instantly affect children’s direct perception. For example, 3-year-old children perceive marked boundaries in their surroundings such as lines, and they may utilize them as a basis for chasing games. On the other hand, if they encounter an immovable boundary, like a wall, they might lean against it to rest (Huang, 2017). Thus, children’s direct perceptions of environments guide their subsequent actions, while the movement creates new opportunities to detect information (Sadler and Given, 2007; Wicker, 1979); therefore, perception and action are functionally inseparable. Interaction with the environment during movement generates new environmental information, ultimately forming a perception–action loop (see Figure 1).

Another core of Gibson’s ecological psychology theory is the concept of affordance, which refers to the opportunities for action that the environment offers to an organism with specific physical abilities (Heft, 2001). For example, a horizontally rigid surface provides support (walkability) for most animals and humans. Affordance is neither a subjective quality nor an objective physical property, but it is a relationship arising from the mutual adaptation between an organism and its environment (Chemero and Turvey, 2007). In Gibson’s theory, affordance is also viewed as information provided by the environment that children directly perceive (Heft, 1989). Fundamentally, the environment provides ecological information in terms of ambient energy arrays, which specify the availability of affordances (i.e., potential affordance). An organism’s capabilities, properties, and ecological niche determine which affordance it perceives and, consequently, acts upon. Organisms thus access ecological information through direct perception and act based on perceived affordances. These interactions between action and ambiance alter affordances and ecological information, forming a reciprocal loop. In essence, Gibson’s ecological theory presents a mutualistic relationship between perception and action in which affordance serves as a bridge connecting the organism and its surroundings.

The ecological psychology approach has been applied to studying the child–environment relationship. For instance, based on Heft’s (1988) functional taxonomy of affordances, van Liempd (2018) developed a specific tool, the SACID tool, to identify potential affordances in the classroom. This tool allows observers to record children’s momentary actions at the spatial level (e.g., table, chair, and floor) in preschool classrooms. The SACID tool has proven useful in Dutch kindergarten classrooms and may be applicable in other European contexts. Nevertheless, it poses challenges for measuring affordances in Chinese preschool classrooms. For example, in the SACID, an adult-height table affords stabilization and climbing opportunities. However, in Chinese kindergartens, children rarely have access to teachers’ areas, making it unlikely they would climb on such tables. From an ecological perspective, the preschool environment offers opportunities for actions related to children’s physical abilities and needs, emerging apparent developmental discrepancies from adults (Sando and Sandseter, 2022). For instance, in China, a low chair provides a surface on which 3-year-old children can sit or move, whereas, for older children aged 5 years, it might serve as a tool for stacking or constructing. Therefore, ecological psychology provides a valuable framework for understanding how preschool children perceive and engage with their environments. Compared to traditional research approaches, studying how children interpret their environments based on affordances holds greater psychological significance (Morgenthaler et al., 2024; van Liempd, 2018). While the immediate perception–action loop is helpful in understanding children’s momentary performances and the environmental features that afford them, it remains challenging to explore the formation and evolution of long-term behavioral patterns in children.

In comparison, Barker (1968) concept of *behavior setting* further converges on the involvement of individuals and their behavior, generating the operations of behavior settings (Heft, 2018). Behavior setting, introduced as a high-level eco-behavioral unit encompassing joint behaviors, has spatiotemporal characteristics that emphasize enduring behavior patterns. Based on the features of behavior setting, Moore et al. (1996) recommended that well-defined center-based childcare facilities should have environmental attributes such as a modified open plan with several distinct “activity pockets.” In a later study, Moore grouped 14 childcare centers into three types based on spatial definition: well-defined, moderately defined, and poorly defined. The findings indicated that spatially well-defined classrooms, with flexible boundaries, adaptable layouts, and varied levels, offered children ample opportunities for exploratory behaviors, cooperation, and social interaction (Moore, 1986). Subsequent empirical studies confirmed that the classroom spatial components enhance functionality, promoting cooperative and social communication among children (Abbas et al., 2016; Maxwell, 2007; Zimmons, 1997). These studies exploited the nested structure of behavior settings to highlight how eco-behavioral units support positive child behaviors—specifically, how certain behavior settings benefit children’s positive comportment. While subsequent research has continued within this paradigm (e.g., Cosco, 2006; Jafari et al., 2020), it has often overlooked the internal dynamics within behavior settings. Researchers have focused on beneficial behavior settings from the perspective of physical environments, leaving open questions about how behavioral patterns form and engage within these settings.

In summary, both affordance and behavior-setting theories focus on the human–environment relationships. However, a transparent distinction between these two theories is that affordance emphasizes immediate perceptions (Chemero, 2018), whereas behavior setting highlights long-term behavior patterns (Heft, 2018). At first glance, these concepts appear contradictory regarding temporal focus. Nevertheless, in the context of Chinese kindergartens, classrooms are used for both teaching and indoor play, unlike in Western contexts such as Britain (Huang, 2017) and the Netherlands (van Liempd et al., 2018). This dual function, combined with flexible layouts and relatively stable peer groups, creates abundant opportunities for children to interact and develop diverse play patterns (Lerstrup and Konijnendijk van den Bosch, 2017). In other words, children’s intricate momentary actions (i.e., affordances) contribute to long-standing patterns of behaviors (i.e., behavior setting) during play sessions in classroom settings. Thus, from an ecological perspective, children’s performances in the classroom serve as a bridge interlinking affordance and behavior-setting concepts. This connection is the primary motivation of the current study. Therefore, the study aimed to explore the feasibility of integrating affordance and behavior-setting theories within an eco-psychological framework, developing a theoretical model for studying the physical environment and children’s behavior in childcare settings. Admittedly, various factors constrain children’s classroom behavior, such as teacher’s strategies of classroom management (Korpershoek et al., 2016) and material placement (Nordtømme, 2012), teacher–child relationship (Mortensen and Barnett, 2015), peer relationships (Karaca et al., 2020), and individual personality differences (van der Graaf et al., 2018). However, there remains a need to examine the role of the physical environment in shaping children’s dynamic behavioral patterns, especially in China’s socio-cultural context. The following section begins with an example and discussion of affordance, focusing on its application in children’s environments.

## 2 Affordance as an ecological approach in children’s environments

From the perspective of the perception–action loop, the affordances of the physical environment in childcare spaces are theoretically ongoing and diverse. Similar to perception guides action, in turn, actions proffers new environmental information to the perception system (Kyttä, 2002). The internal dynamics of affordance specifically interpret the perception–action loop, emphasizing both perception and utilization (Sando and Sandseter, 2022). Furthermore, kindergarten-aged children are selected as the study’s subjects because socio-cultural factors have not yet fully shaped their behavior (Koch et al., 2011). In other words, their imagination and creativity drive them to explore their surroundings, offering numerous opportunities for engaging with the environment (Karaca et al., 2020). For instance, an empty zip-top might be seen by adults as recyclable waste or a component for making handicrafts. However, for a kindergarten child, it could become a football in a game or an evil monster in a fantasy play session. Thus, from a child’s perspective, interaction with physical environments involves exploiting material elements in their surroundings to realize their actions (Bateman and Church, 2017). In this process, children experience the process of direct perception, utilization, and shaping of objects through their actions.

Kyttä (2003) conceptualized this process as the dynamics of affordances: perceived, utilized, and shaped affordances. In her model, three levels of affordances and potential affordances within the environment are in constant interchange and circulation. However, the levels of affordances used in later studies have shown significant socio-cultural attributes (e.g., Aziz and Said, 2016; Sando and Sandseter, 2022; Sandseter et al., 2022), leading to the dilemma of replicating within the Chinese kindergarten context. The layer of socio-culturally preferred affordances (Loveland, 1991), meanwhile, highlights the limitations imposed by social and cultural factors on affordance theory. Therefore, exploring how children perceive their surroundings and communicate within different socio-cultural settings is both rational and necessary. The following sections will discuss research on affordances from the perspectives of the dynamics and functional properties of affordances in the context of Chinese kindergarten indoor environments.

### 2.1 Dynamics of affordances

The notion of layers of affordances (Loveland, 1991), introduced from a macro perspective, theoretically aids researchers in distinguishing the effects of various environmental affordances on human behavior. However, kindergarten classrooms, as concrete environments, afford children’s curiosity-driven exploration, creativity, and imagination in relation to their surroundings (Barnikis, 2015; van Liempd et al., 2018), resulting in unpredictable behaviors in real-world scenarios. Hence, considering only the perspective of affordances layers to analyze a childcare classroom environment neglects the fluidity and interconnectivity among children, peers, and surroundings.

In line with the framework refined by Kyttä (2003), which builds on Heft’s (1988) original model, affordances can be separated into two facets. The first aspect, potential affordances, refers to environmental qualities, while the second is the relationship between individuals and the environment. Specifically, the affordances actualized by individuals through perception, utilization, and shaping. Potential affordances, defined as the functional properties provided by the environment, are theoretically infinite (McLaren et al., 2011), as individuals only use a subset of these affordances within one object (Kyttä, 2003). In other words, potential affordances and actualized affordances represented environmental and human factors, respectively, in the organism–environment system. However, in contemporary early education settings in China, several influential factors constrain potential affordances, such as teacher’s philosophy (Li et al., 2014; Li et al., 2019), same-age classroom strategies (Wu et al., 2022), and spatial components in the classrooms (Acer et al., 2016; van Liempd et al., 2018).

From a physical perspective, the design and layout of space (Chawla et al., 2011), including the availability of activity areas (Cosco, 2006; McKenzie et al., 2000) and the amount of open space (Benchekroun et al., 2020; Huang, 2017), offer spatial affordances that provide varied opportunities for children’s movement and activities (Cosco, 2006; Fusco, 2016). For example, in a study examining the play preferences of Chinese kindergarten children, 60% spent no more than 15 min in the role-playing area, while 66.9% stayed over 30 min in the operating area (Ma and Hao, 2024). Moreover, boys tend to be more active in open spaces, while girls prefer to play in corners (Luo, 2023). Even in open spaces without designated play areas, children, particularly those aged 3–4 years, engage in free play (Huang, 2021). Furthermore, the arrangement of different types of play materials also influences children’s play preferences. For instance, high-structured play materials enhance the constructive intent of 5–6-year-old children, while low-structured toys offer flexibility for creativity (Dong, 2007). Thus, the potential affordances in China’s kindergarten classrooms exist in both teacher-organized activity areas and open spaces without play materials. However, these studies overlooked affordances related to other functional components (e.g., table, chair, and floor) within the classroom environment. Research by van Liempd et al. (2018) indicates that the depth of children’s spatial exploration is positively correlated with table use. While this study focuses on Dutch children aged 1–4, its findings still provide valuable insights for this study. Consequently, examining the influence of stationary or ambulatory compositions in classrooms on children’s actions, particularly for those in the 4–6 age range, may reveal differences in potential affordances between Western and non-Western kindergarten environments regarding spatial components.

Furthermore, when children engage in the childcare environment, the dynamic process of perception and action actualizes affordances (Kyttä, 2003). Similarly, in the perception–action loop of ecological psychology, children utilize their environment by perceiving its potential affordances and shaping them into distinctive ones. This process of perceiving, utilizing, and shaping the environment comprises the three levels of actualized affordances (Kyttä, 2002). From their perspectives, children may alter the intended functions of certain objects or environments through interactions (e.g., using a crayon as a sword). In this context, they interact with peers and unintentionally develop social skills and competencies (Huang, 2017; van Liempd, 2018). As Kyttä introduced the dynamics of affordances, researchers have exploited this concept to examine children’s indoor play behavior in childcare settings. Sando and Sandseter (2022) conducted structured interviews with 71 Norwegian children aged 3–6 years to explore how early childhood education and care (ECEC) environments afford construction, pretend, and physical play. Their study found that loose materials provided children with more play options, aligning with Heft (2003) and Nicholson (1971) views. Sandseter et al. (2022) further identified a strong correlation between children’s construction play and tables sized for both children and adults. However, these studies focus primarily on objects as affordances for play behavior, with limited attention to children’s direct perceptions of their environment (e.g., tables) and immediate actions on these elements. In other words, constructive play as an outcome has been extensively discussed in studies of China (e.g., Ma and Hao, 2024; Luo, 2023) and other countries (e.g., Sandseter et al., 2022; Storli and Hansen Sandseter, 2019) Nevertheless, the transactional process between children and spatial components remains underexplored. This omission indicates that many studies applying affordance theory have focused on interpreting children’s environmental engagement through play behaviors, rather than examining direct perception of environmental factors to assess environmental influence on their behavior.

Additionally, interviewing children relies heavily on the interviewer’s experience and judgment, which may affect the reliability and validity of responses from kindergarten-aged children compared to direct observation. As such, the majority of studies on children’s behavior employ field observation methods (e.g., van Dijk-Wesselius et al., 2022; van Liempd et al., 2018; Sandseter et al., 2021). Furthermore, adult-imposed rules also influence children’s behaviors in actualizing affordances (Sando and Sandseter, 2022). Hence, when analyzing Chinese kindergarten children’s actions, it is essential to consider whether teachers or caregivers impose restrictions on children’s free play. In ecological psychology, analyzing children’s actualized affordances requires examining their immediate actions, rather than long-term play behaviors, to capture the perception–action loop (Raymond et al., 2017). The taxonomy of functional properties of environmental affordances offers a framework to summarize the relationship between children’s real-time actions and environmental features.

### 2.2 Functional taxonomy of affordances

The hierarchy of affordances provides a breadth dimension for related studies, while the functional taxonomy of affordances delves into depth. In 1988, Heft reviewed several studies on children’s outdoor activities through the lens of affordances, identifying various functional properties linked to outdoor environments. He introduced a preliminary functional taxonomy of children’s outdoor environments through descriptive analysis, categorizing affordances into 10 subsets. Briefly, Heft’s taxonomy encompasses four main themes: body-scale affordances, surface affordances, object affordances, and environmental affordances. Body-scale affordances pertain to competencies and dimensions directly related to an individual’s body, such as jumping, climbing, or reaching. Surface affordances relate to the properties of a surface, such as whether it can be walked or slid upon. Object affordances concern traits and functions, such as throwing or grabbing, while environmental affordances relate to overall layouts or attributes, such as shelter, exploration, and navigation. These affordances represent potential functions offered by the environment, constrained by socio-cultural factors, the material environment, and individual attributes (Lobo et al., 2018). However, Heft (1988) functional taxonomy, rooted in children’s outdoor activities, emphasizes a strong reliance on natural environments and elements. In Chinese kindergarten classrooms, which largely consist of artificial objects, some affordances are less applicable. For instance, smooth slopes or climbing structures are uncommon in classroom spaces. Therefore, subsequent researchers developed more refined taxonomies to capture diverse affordances across different environments (Huang, 2017; Kyttä, 2003; McLaren et al., 2011; Rietveld and Kiverstein, 2014; Morgenthaler et al., 2024).

Kyttä (2002) conducted a survey on children’s outdoor environments across urban, suburban, town, and rural areas in Finland and Belarus, using interviews to explore children’s activities, play experiences, and environmental affordances. Building on Heft (1988) work, Kyttä expanded the functional taxonomy by incorporating social affordances. Huang (2017) further refined this approach within British kindergarten contexts, distinguishing between environmental factors and affordances for individual and social behaviors. Additionally, van Liempd (2018) developed a detailed classification of indoor furniture, flooring, decorations, fences, and activity areas in childcare centers, with spatial components as a focal point. However, Kyttä’s taxonomy touches only briefly on “environmental opportunities for sociality,” without detailing the connection between social affordances and environmental functions. While Huang’s research differentiates between affordances for solitary and social behaviors, it lacks a comprehensive categorization for indoor environments, limiting social behavior affordances to shared use and conflict. Although van Liempd’s tools specifically target childcare center interiors, he overlooks how social interactions between children and play materials influence affordances.

Overall, factors shaping affordances include animate and inanimate elements (Aziz, 2014), natural (Herrington and Brussoni, 2015; Prins et al., 2022; Rietveld and Kiverstein, 2014), artificial (Fusco, 2016; Atmodiwirjo, 2014; Yusof et al., 2022), physical (Heft, 1988; van Liempd, 2018; Sando and Sandseter, 2020), and social (Huang, 2017; Kyttä, 2002) factors. Although artificial materials dominate indoor kindergarten environments, previous research primarily classifies affordances based on outdoor contexts, supporting the argument for children’s independent mobilities (Kyttä, 2003) and natural elements (Laaksoharju et al., 2012; Zwierzchowska and Lupa, 2021; Zamani, 2017). While natural elements offer developmental benefits, indoor activities remain crucial in the Chinese pedagogical context, where outdoor activities are limited by seasonal and climatic conditions. Consequently, affordance taxonomies for Chinese kindergarten classrooms should consider not only basic physical components (e.g., tables, chairs, shelves, and floors) but also differences in children’s use of play materials. For example, Huang (2021) found through a survey of Shanghai kindergartens that children aged 3–4 prefer manipulating objects, while those aged 4–5 focus more on constructing objects. Thus, differentiating play materials is essential in developing an affordance taxonomy suited to China. Additionally, movable and stationary objects or boundaries should be separately addressed.

To this extent, this section discusses the dynamics and functional taxonomy of affordance theory based on empirical studies of children’s environments and behaviors. From the perspective of ecological psychology, children’s instantaneous actions reflect their direct perception of their surroundings. However, as Chinese kindergartens represent a continuous 3-year stage, immediate actions alone cannot fully capture the development of children’s play patterns. The concept of behavior settings offers insight into the progression of collective behaviors and long-term behavior patterns in specific spaces, complementing the perception–action loop in affordance theory. The following section will inquire into behavioral setting theories related to children’s environments.

## 3 Behavior setting theory in children’s environments

Although behavior setting theory does not prioritize direct perception, certain elements intersect with key principles of ecological psychology. Originally introduced and defined by Barker et al. in research conducted from 1947 to 1972 (e.g.,^9003,5^), behavior setting theory has since been widely applied in subsequent studies on human–environment relationships across the world (Heft, 2018; Keshmiri and Nikounam Nezami, 2023; Wicker, 1979, 2012). By observing children’s behaviors at various locations within communities, Barker discovered that their actions were closely linked to specific places, a phenomenon central to the behavior-setting concept (Barker, 1978). In the initial formulation of the theory (Barker, 1963), a behavior setting was characterized as a spatiotemporal locus with clear boundaries. It is a nested structure that arranges various fixed entities and events and is independent of subjective perception. In particular, individuals’ actions in a peculiar behavior setting seem predictable based on these features. Barker (1968) later elaborated that a behavior setting possesses not only temporal and spatial dimensions but also includes at least one consistent pattern of behavior and a milieu, which are structurally synomorphic. Within this embedded structure, the milieu objectively surrounds behavior, remaining independent of individual perceptions. Additionally, milieu and behavior are integrated within the behavior setting, exhibiting a high degree of interdependence.

A behavior setting, composed of the milieu and a standing pattern of behavior with attributes of synapomorphy, interdependence, and circumjacency, formally emerged as a concept in psychology. Wicker (1987) labeled behavior settings as socially constructed entities, analyzing them in terms of resources, internal dynamics, and context. Fuhrer (1990) reinforced Wicker’s definition and further provided abstract descriptions of behavior settings in the dimension of social psychology. Hence, a behavior setting encompasses not only individual actions but also collective behaviors, reflecting dynamic internal structures. In summary, the concept of behavior setting begins with the study of simple units of everyday behavior in a sample of children, followed by an interest in accenting its internal dynamics and evolution, ultimately shifting toward the realm of subjective experience. Therefore, overall, behavior setting has two core properties, namely, structural and internal dynamics, and is compounded by the milieu and standing pattern of behaviors. The content of the following subsections will be encircled with these attributes.

### 3.1 Nested structure of behavior setting

From a constitutive sight, a behavior setting is similar to a genotype, which is a high-level dynamic environmental unit or an eco-behavioral unit comprising individuals’ joint actions and material features of the location (i.e., milieu; Heft, 2018). The physical elements within the environment offer opportunities for both solitary and collective action patterns, largely aligning with Gibson’s concept of affordances. In this study, “milieu” refers to items such as desks, chairs, foam pads, decorations, walls, floors, and stairs in kindergarten classrooms, which collectively provide opportunities for various behaviors among children. The milieu exists objectively, as a subordinate component distinct from the broader material environment, and provides potential ecological resources for individuals’ actions (Giusti et al., 2014). This concept aligns closely with potential affordances, forming part of the rationale for integrating behavior setting and affordance theories in this study.

While the milieu emphasizes spatial attributes within behavior settings, the standing pattern of behavior highlights temporal attributes. For instance, when individuals enter a behavior setting and engage in a series of actions, these behaviors are not considered a standing pattern, unless they are sustained over time. Only when individuals’ joint actions persist does a behavioral pattern become established (Heft, 2001). This assertion contrasts with the immediate focus on individual actions in affordance theory. Many studies on behavior settings, however, have emphasized spatial features over temporal continuity. For example, Cosco (2006) identified sand pits, triangular areas, and grassy spaces in children’s outdoor environments as behavior settings. Similarly, Refshauge et al. (2013) classified settings such as slides, playrooms, and volleyball courts. These studies often overlook the standing pattern of behavior—ongoing, collective actions within the milieu. The formation of a standing pattern relies on repetitive behaviors, sustained duration, and steady frequency as participants interact with physical entities. Thus, a behavior setting comprises both a milieu, rich in material attributes, and standing patterns of behavior, which are consistent and sustained episodes. Consequently, behavior setting, milieu, and standing behavior patterns are structurally synomorphic.

Viewing a classroom or playground as a behavior setting oversimplifies the concept, as true behavior settings involve enduring interaction among individuals within the environment. For example, a football field is not a behavior setting on its own; it becomes one through the continuous actions of players, referees, coaches, and spectators interacting with the stadium’s physical elements. Similarly, childcare classrooms, as environments with multiple behavioral settings, are expected to facilitate encounters among children and interactions with material surroundings, allowing children to shape these settings over time.

### 3.2 Internal dynamics of behavior setting

A behavior setting operates as a dynamic mechanism with a life cycle, arising, developing, and sometimes dissipating (Luke et al., 1991). Its internal dynamic cycle is driven by the interplay between milieu and standing behavior patterns. The milieu, whether natural or artificial (Popov and Chompalov, 2012), provides objective material features, but without participant interaction, it remains inactive (Heft, 2018). The reciprocal engagement between participants and the physical environment forms standing behavior patterns, which, in turn, shape or self-regulate the milieu (Wicker, 1987). While both behavior setting and affordance theories discuss human–environment interactions, behavior setting theory emphasizes continuous interaction between behavior and environmental factors, treating human and environmental components as equally significant (Heft, 2018). In contrast, affordance theory prioritizes human perception and action, placing less emphasis on environmental factors (Raymond et al., 2017). This discrepancy explains why studies on early childhood environments often focus on children’s behaviors and perceptions, rather than on design criteria for physical environments. This study aimed to integrate behavior setting and affordance theories to address this gap.

Furthermore, the dynamics of affordance suggest a continuous cycle, implying that as long as organisms and environments coexist, the environment theoretically provides perpetual support for human actions. For instance, even when someone sits on a sofa and enters a trance, the sofa affords the possibility of sitting and trancing. However, this scenario does not constitute a behavior setting, as it lacks the joint behaviors of multiple individuals, and momentary actions cannot establish long-term behavioral patterns. Unlike the theoretically infinite cycle of affordances, the dynamics of a behavior setting begin with the interaction between milieu and behavior and evolve through ongoing exchanges. Over time, some behavioral patterns may diminish, leading to the decline of the setting. The primary criterion for a behavior setting is the threshold of interdependence between its elements (Barker, 1968).

In kindergarten classrooms, more specifically, determining the interdependence between children’s behaviors and the classroom milieu involves examining their mutual influence. Environmental factors, such as size, noise, and temperature, along with spatial features and components, affect behavior. Children’s actions can be synthesized through video recording (Huang, 2017), interval sampling (Jin, 2018; Rubin, 2001), and behavior mapping (Cosco et al., 2010; Cox et al., 2018). The predictability of the environment’s impact on behavior reflects this interdependence. Therefore, in this study, classrooms are seen as clusters of sub-settings, such as teacher-directed settings for instruction, dining, and resting, and partially child-directed areas for flexible play. The interaction among material elements, children, peers, and the locale generates social events, play behaviors, and episodes of social play, shaping both the cluster setting and its sub-settings. Empirically, this leads to the emergent process of a dynamic, collective inter-individual unit—what is known as a behavior setting.

In Chinese kindergartens, spatial settings are more flexible than in Western playrooms, often adapting to teacher-directed resource allocation (Ma and Hao, 2024; Dong, 2007). Chinese children also tend to exhibit collective behaviors, differing from Western social patterns. Current research on Chinese kindergarten environments has primarily examined children’s play behavior patterns (Han et al., 2023), layout (Cai et al., 2024), and play materials (Ye, 2015), revealing a significant gap in studies on dynamic behavior settings in classrooms. Similarly, this phenomenon occurs in Western studies, where behavior settings are often treated as static, neglecting their internal dynamics (e.g., Refshauge et al., 2013). Thus, investigating dynamic behavior settings in children’s indoor environments through empirical research is essential.

## 4 Findings and conclusion

The current theoretical framework delineates approaches to affordance and behavior setting within ecological psychological sight. Both frameworks fundamentally explore the dynamic relationship between individuals and the environment, though their focuses differ. Affordance theory emphasizes individual perceptions and uses of the environment, whereas behavior setting theory highlights enduring interactions between individuals and the milieu. Ecological psychology, as informed by previous studies, offers a people-centered perspective for researching children’s environments and behaviors. However, recent studies tend to concentrate on either the affordance side, focusing on children’s perception–action loop (Barrable and Barrable, 2024; Sando and Sandseter, 2022), or on the synomorphy between individuals and their surroundings, related to human wellbeing (Jafari et al., 2020; Keshmiri and Nikounam Nezami, 2023). Consequently, there is a lack of studies that integrate the primary components of affordance and behavior setting within ecological psychology to examine children’s actions and their contexts.

**Figure 2:**
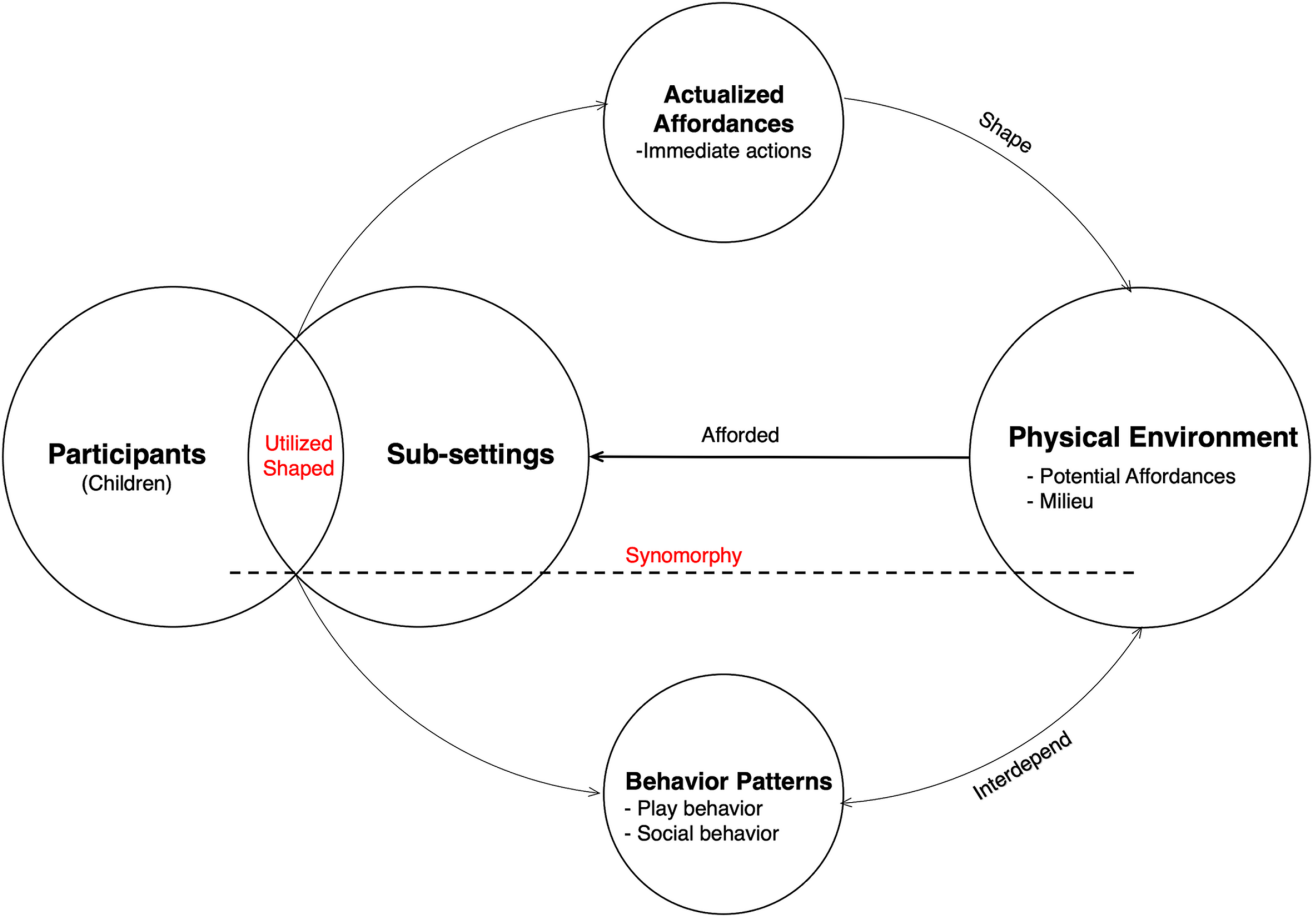
Integrating the conception of affordance and behavior setting into a theoretical framework for the research on the physical environment of preschool classrooms and children’s behavior.

Following a review of previous studies within ecological psychology, this research integrates affordance and behavior-setting traits to construct a theoretical framework (see Figure 2). Behaviorally, this framework examines children’s immediate actions as responses to perceived environmental features, explores correlations between actions and behavior segments, and identifies the transition from behavior episodes to behavior patterns. Environmentally, it investigates the support provided by technological norms, spatial characteristics, and physical components in classrooms for children’s actions, play, and social interactions. Methodologically, participatory and non-participatory systematic observation, interval sampling (e.g., 5-s interval, 5-min episode), and behavior mapping facilitate effective data collection and analysis of children’s actions. Additionally, spatial deconstructing (Huang, 2017) and rating scales (Harms et al., 2014; Moore, 1994; Moore and Sugiyama, 2007) (e.g., Moore’s CPERS/ECPES, Maxwell’s ICRS, and ECERS) aid in gathering data on the physical classroom environment. This framework helps explore how physical environmental factors influence the formation and modification of children’s play behavior patterns in classrooms. However, its limitation lies in the predominant focus on physical environmental factors on children’s autonomous play behavior, potentially overlooking the impact of teachers, caregivers, and parents on children’s behavior.

Theoretically, the classroom environment offers potential functions and elements that form the classroom’s cluster setting and sub-settings. Children, as participants, perceive these functions and convert them into perpetual actions with peers and material properties, thereby actualizing these affordances to maintain the operation of the sub-settings. Meanwhile, as children use and shape their environment, a standing pattern of behavior—comprising play and social interactions—emerges. In this sense, children’s behavior patterns are expected to be highly interdependent with material environmental elements, fostering synapomorphy between participants and the physical environment. Ultimately, the attainment of the synapomorphy of children and the environment has led to a child-friendly atmosphere in the classroom.

This theoretical framework offers guidance for future research on children’s indoor environments, especially within China’s Early Childhood Education and Care context. Thus, future studies should examine the nexus within this framework through a field investigation of the physical environments of childcare facilities and observation of children’s actual behaviors in their free play sessions to ascertain whether their involvement is congruent. In addition, this framework considers utilized and shaped affordances as the process of actualized affordance. Further studies should converge on children’s perceptual progress in environmental attributes and the nexus of their play preferences and actions.

## Data Availability

The original contributions presented in the study are included in the article/supplementary material, further inquiries can be directed to the corresponding author.
